# Safety of combination antiretroviral prophylaxis in high-risk HIV-exposed newborns: a retrospective review of the Canadian experience

**DOI:** 10.7448/IAS.19.1.20520

**Published:** 2016-02-12

**Authors:** Fatima W Kakkar, Lindy Samson, Wendy Vaudry, Jason Brophy, Jean-Baptiste Le Meur, Normand Lapointe, Stanley E Read, Ari Bitnun

**Affiliations:** 1 Department of Pediatrics, CHU Sainte-Justine, Université de Montréal, Montréal, QC, Canada; 2 Department of Pediatrics, Children's Hospital of Eastern Ontario, University of Ottawa, Ottawa, ON, Canada; 3 Department of Pediatrics, Stollery Children's Hospital, University of Alberta, Edmonton, AB, Canada; 4 Department of Social and Preventive Medicine, Laval University, Québec City, QC, Canada; 5 Department of Pediatrics, Hospital for Sick Children, University of Toronto, Toronto, ON, Canada

**Keywords:** Prevention of mother-to-child transmission, HIV, neonatal prophylaxis, safety, combination antiretroviral therapy

## Abstract

**Introduction:**

The optimal management of infants born to HIV-positive mothers who are untreated or have detectable viral load prior to delivery remains controversial. Despite the increasing use of combination antiretroviral therapy (cART) for post-exposure prophylaxis (PEP) of neonates at high risk of HIV infection, there is little safety and pharmacokinetic data to support this approach. The objective of this study was to evaluate the safety and tolerability of cART for PEP in HIV-exposed neonates.

**Methods:**

Retrospective study on 148 cART and 145 Zidovudine (ZDV) monotherapy-exposed infants identified from four Canadian centres where cART for PEP has routinely been prescribed in high-risk situations. Physician-reported adverse events and clinical outcomes were extracted by chart review. Haematological and growth parameters at birth, one and six months of age were compared between cART and ZDV-exposed infants using multivariate mixed effects modelling.

**Results:**

Non-specific signs and symptoms were reported in 10.2% of cART recipients versus none of the ZDV recipients. Treatment was discontinued prematurely in 9.5% of cART recipients versus 2.1% of ZDV recipients (*p=*0.01). In the multivariate model, cART recipients had lower mean haemoglobin (decrease of 2.07 g/L) over the 6-month period compared with ZDV recipients (*p=*0.04), but no effect was seen on absolute neutrophil count. cART recipients had lower weight and smaller head circumference at birth and one month of age compared with ZDV-exposed infants; these differences were no longer significant at six months of age.

**Conclusions:**

cART administered at treatment doses for PEP in neonates was generally well tolerated, though a higher incidence of non-specific signs and symptoms and early treatment discontinuation occurred among cART recipients.

## Introduction

While the overall risk of perinatal HIV transmission in the developed world has decreased to less than 1%, transmission still occurs, most often due to failure to diagnose HIV in pregnant women in a timely manner, or because of poor maternal drug adherence to, and/or refusal of, antiretroviral therapy [1]. Despite the relatively high risk of transmission in these situations, the optimal management of infants born to HIV-positive mothers, who are untreated or have detectable viral load prior to delivery despite receiving antenatal therapy, remains controversial. Current US Department of Health and Human Services (DHHS) guidelines recommend a six-week course of Zidovudine (ZDV), along with three doses of Nevirapine (NVP) in the first week of life for infants born to mothers who have not received any treatment [2]. However, in practice, many paediatric HIV specialists prescribe various combinations of three or more drugs to such “high-risk” neonates. In a national web-based survey of US care providers between December 2009 and January 2010, 62% reported the use of combination antiretroviral therapy (cART) during the previous year [3]. In a recent study from the United Kingdom, cART was prescribed to 71% of infants born to untreated mothers [4].

Despite the increasing use of cART for prophylaxis in neonates at high risk of HIV infection, there is limited data on the safety of these different combination regimens, or on the appropriate dosing for the antiretroviral (ARV) medications used for this purpose. Transient haematological toxicity has been reported with the use of six-week post-natal ZDV [5], the recommended regimen in standard-risk situations, and a number of studies have suggested increased haematological toxicity associated with the addition of a second nucleoside analogue [6, 7]. Neither the safety nor the optimal dosing of additional drugs used for prophylaxis in neonates (non-nucleoside reverse transcriptase inhibitors or protease inhibitors (PIs)) has been well defined [8–13]. Furthermore, there is emerging concern about the long-term impact of increased ARV exposure on the growth of exposed infants [14, 15].

In four Canadian centres, triple cART at treatment doses has been routinely prescribed for the post-exposure prophylaxis (PEP) of neonates deemed at high risk of HIV infection, either because of detectable maternal viral load near delivery or in the absence of viral load results, clinical factors such as poor adherence or late diagnosis. In standard-risk situations, six weeks of ZDV monotherapy is prescribed. Given the limited data to support the use of cART, the primary objective of this study was to compare the safety and tolerability of cART versus ZDV monotherapy of PEP with respect to adverse events, haematological and growth parameters in exposed neonates.

## Methods

HIV-1 exposed children were eligible for this study if they were started on treatment doses of cART within 72 hours of birth because of incomplete maternal virologic suppression at delivery or, in the absence of maternal viral load results, a maternal history of incomplete adherence or non-adherence to cART, or late pregnancy initiation of cART. Neonatal cART was defined as any regimen of three or more antiretroviral agents, prescribed at treatment doses, for a minimum of six weeks. Regimens included ZDV (2 mg/kg every six hours prior to 2011, and 4 mg/kg twice daily in 2011–2013) for six weeks, Lamivudine (3TC) (2 mg/kg twice daily) for six weeks, with the addition of either NVP (150 mg/m^2^ daily for 14 days, followed by 150 mg/m^2^ twice daily for 14 days) or Nelfinavir (NFV) 40–50 mg/kg twice daily for six weeks) or LPV/Ritonavir (LPV/r) (300 mg/m^2^ twice daily) for six weeks. Combination regimens containing single or three doses of NVP were excluded.

Subjects were identified for the period 1997–2013 from the clinical databases of four paediatric HIV-care institutions in Canada: The Hospital for Sick Children, Toronto; Children's Hospital of Eastern Ontario, Ottawa; Centre Hospitalier Universitaire (CHU) Sainte-Justine, Montreal; Stollery Children's Hospital, Edmonton. A control group consisting of all infants of HIV-positive mothers who were prescribed ZDV monotherapy during a three-year period (2010–2012) at the Hospital for Sick Children was selected (*n=*148). Approval for the study was obtained by the ethics review board of each participating institution; given the retrospective nature of the study, individual patient consent was not deemed necessary by the respective ethics review boards for the chart review.

### Statistical analysis

Baseline characteristics were reported as means with standard deviation (SD) for continuous variables and proportions for categorical variables. Comparisons between cART- and ZDV-exposed infants were performed using chi-square tests or Wilcoxon rank-sum. Haemoglobin, neutrophil count and growth parameters (head circumference (HC), weight and length) of cART- and ZDV-treated infants were compared at one, two and six months of age (haematological parameters) and at birth, one and six months of age (growth parameters) by Student's t-test or Wilcoxon rank-sum where appropriate. For the the three-way comparison between PI based-cART, NVP based cART and ZDV groups, the Kruskal-Wallace test was used.

Multivariate mixed models, allowing for repeated measures, were constructed to determine the overall effect of treatment category on haematological and growth parameters. For haematological parameters, effect of treatment category was adjusted for chronological age (variable for first, second or third visit corresponding to one, two and six months of age given the natural change in these parameters over time), gestational age and ethnicity (neutrophil count only). Due to a large number of missing values for haemoglobin and neutrophil count at baseline, these parameters were not included in the models. For growth parameters, effect of treatment was adjusted for chronological age, gestational age, gender and birthweight. All infants were included in the analysis of haematological toxicity and growth at one month; for all subsequent analyses infants confirmed to be HIV positive (*n=*13) were excluded from the analysis as all were maintained on ARV drugs. The analysis was performed using SAS statistical software, version 9.2 (SAS Institute, Cary, NC, USA).

## Results

One hundred and forty-eight infants received triple cART during the study period. NFV-based cART was the most common regimen (55%) followed by NVP-based cART (40%) and LPV/r-based cART (5%). The most common reason for prescribing cART was documented detectable maternal viral load (78.4%). Other overlapping factors included poor adherence (45%), late diagnosis (20%), no antenatal care (8%) and refusal of antenatal ARV therapy (8%). Fifteen of the cART-treated infants and five of the ZDV-treated infants had missing six-month data, either because they were lost to follow-up (*n=*8) or they did not attend the scheduled six-month visit (but were seen at 18 months of age or older) (*n=*12).

Baseline maternal and infant characteristics according to treatment group are described in Table 1. Mothers of cART-treated infants were younger, had higher viral loads and lower CD4 counts at the time of delivery, and were more likely to deliver vaginally and prematurely than mothers of ZDV monotherapy-treated infants.

**Table 1:** Baseline demographic and clinical characteristics of mothers and infants^a^

	cART (*n=*148)	Zidovudine (*n=*145)	*P*^b^
Maternal age (years)	29.3±5.0	33.7±5.1	<0.0001
Maternal viral load (c/mL)	11,478±35,351	<40	<0.001
Maternal CD4 count (cells/µL)	411±258	519±220	0.0008
Timing of diagnosis, *N* (%)			
Prior to pregnancy	91 (61.9)	128 (88.3)	
During pregnancy	51 (34.7)	17 (11.7)	
At delivery	5 (3.4)	0 (0)	<0.0001
Mode of delivery, *N* (%)			
Vaginal	56 (37.8)	88 (60.7)	
C/section: Elective	69 (46.6)	39 (26.9)	
C/section: Emergency	23 (15.4)	18 (12.4)	<0.0001
Infant sex, *N* (% female)	73 (49.3)	79 (54.5)	0.41
Gestational Age, Weeks	37.7±2.62	38.4±1.94	0.007
Premature (<37 weeks), *N* (%)	31 (21.0)	18 (12.4)	0.06

a Reported means and standard deviation;

b Chi Square test of proportions or Wilcoxon rank-sum.

**Table 2:** Hematological and Growth Parameters by treatment group†

	Any cART vs. ZDV monotherapy					NVP-based cART vs. PI-based cART vs. ZDV monotherapy			
		
	cART	n*	ZDV	n*	P‡	NVP	PI	ZDV	p‡
Hemoglobin (g/L)									
1 month	105±19	130	109±17	140	0.04	107±21	104±17	109±17	0.09
2 months	104±12	123	105±9	139	0.31	104±11	104±12	105±9	0.58
6 months	118±11	106	121±8	129	0.15	124±9	115±10	121±8	<0.001
Absolute neutrophil count									
1 month	1.79±1.01	128	1.75±0.97	139	0.94	1.74±1.11	1.82±0.93	1.75±0.97	0.66
2 months	1.91±1.40	122	1.91±1.42	139	0.71	1.96±1.29	1.88±1.47	1.91±1.42	0.84
6 months	2.09±1.24	105	2.04±1.38	128	0.37	2.31±1.26	1.98±1.22	2.04±1.38	0.22
Head Circumference (cm)									
Birth	33.2±2.4	111	33.6±2.3	85	0.16	32.5±2.96	33.5±1.98	33.6±2.3	0.03
1 month	36.6±1.5	127	37.2±1.3	141	0.001	36.5±1.6	36.7±1.5	37.2±1.3	0.05
6 month	43.7±1.8	107	44.0±1.7	119	0.13	43.3±2.0	43.9±1.6	44.0±1.7	0.1
Weight (Kg)									
Birth	2.91±0.60	148	3.09±0.56	144	0.01	2.79±0.67	2.98±0.54	3.09±0.56	0.01
1 month	3.91±0.63	134	4.17±0.59	142	<0.001	3.94±0.69	3.89±0.59	4.17±0.59	<0.001
6 months	8.03±1.12	108	8.25±1.12	119	0.14	8.13±1.15	7.97±1.10	8.25±1.12	0.12
Length (cm)									
Birth	49.0±3.6	105	50.2±3.8	80	0.03	48.3±4.7	49.4±2.7	50.2±3.8	0.05
1 month	51.7±2.8	131	52.3±2.7	142	0.004	51.7±3.2	51.6±2.5	52.3±2.7	0.04
6 months	67.1±2.9	107	68.1±3.2	119	0.01	67.5±3.0	66.8±2.8	68.1±3.2	0.03

† Reported means and standard deviation

‡ Student T test, Wilcoxon rank sum, or Kruskal-Wallace test where appropriate.

* n^*^ observations recorded for each parameter out of a total of 148 cART recipients at birth, and 145 ZDV recipients.

Thirteen of the cART recipients were perinatally infected with HIV (8.8%). None of the ZDV recipients were infected. Five of the 13 (38.5%) had HIV-1 detected by PCR within 48 hours of birth suggesting *in utero* infection according to diagnostic standards. For the remaining eight, the timing of infection could not be ascertained as initial testing took place after 48 hours of life. Of the 13 infected children, four achieved long-term sustained virologic suppression, the details of which have been described elsewhere [16].

Non-specific signs and symptoms, including vomiting, diarrhoea, rash, jitteriness or irritability, potentially attributable to medication-related adverse effects, were reported in 10.2% of cART recipients and none of the ZDV recipients (*p<*0.001). ARV treatment was discontinued prematurely in 9.5% (*n=*14) of cART recipients (five NVP-treated and nine PI-treated infants) compared with 2.1% (*n=*3) ZDV recipients (*p=*0.01). Reasons given for premature treatment discontinuation among cART recipients included significant anaemia (*n=*3; haemoglobin at two, four and four weeks of 62, 76 and 92 g/L, respectively), neutropenia (*n=*1; neutrophil count 0.4 cells/mm^3^ at four weeks), non-specific symptoms including rash, vomiting, and diarrhoea (*n=*6), and parental decision (*n=*4). In the six cases of non-specific symptoms, treatment discontinuation was precautionary, as the treating physician indicated that the symptoms were most likely not medication related. In four cases of parental discontinuation, parents stopped the medication without evidence of toxicity or consultation with their healthcare provider. ZDV monotherapy was stopped early in three children, all because of significant anaemia (haemoglobin at four weeks of 76, 85 and 72 g/L).

**Figure 1:**
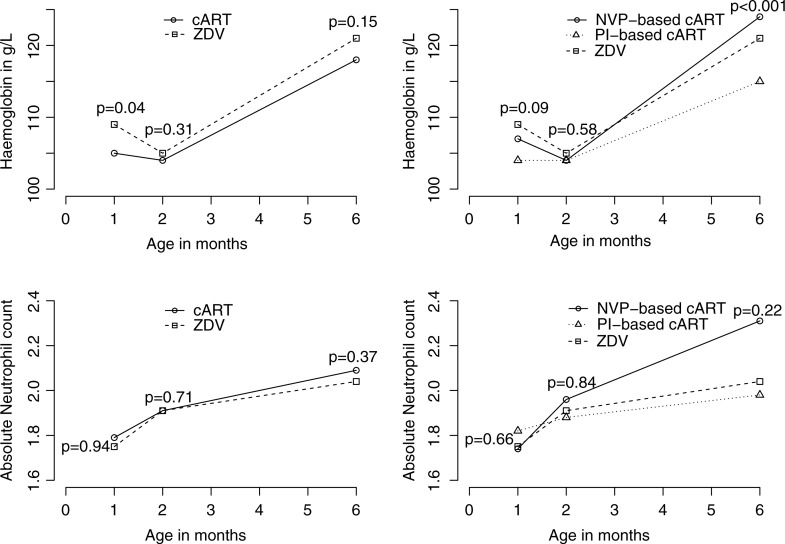
Haematological parameters according to treatment group. **Graphic representation of univariate analyses (Student's t-test, Wilcoxon rank-sum, or Kruskal-Wallace test where appropriate.)**

The potential impact of cART on haematologic parameters was evaluated by comparing haemoglobin level and absolute neutrophil count for cART-treated and ZDV monotherapy-treated infants at one, two and six months of age (Table 2). In the unadjusted analysis, haemoglobin was lower in cART-treated infants compared with ZDV-treated infants at one month of age (mean 105±19 vs. 109±17 g/L, *p=*0.04), but not at two or six months of age (mean 104±12 vs. 105±9 g/L, *p=*0.31 and 118±11 vs. 121±8 g/L, *p=*0.15). In a subgroup analysis of specific treatment type, mean haemoglobin was lower among PI-treated infants compared with NVP-treated infants at one month of age (104±17 vs. 107±21, *p=*0.09), though only statistically significant at six months of age (mean 115±10 vs. 124±9 g/L, *p<*0.001) (Figure 1).

To study the overall effect of treatment type on haemoglobin over time, we constructed a multivariate model adjusting for chronological age and gestational age (Supplementary Table, not shown). Overall, the mean haemoglobin was 2.07 g/L lower among cART versus ZDV-treated infants (*p=*0.04) over the six-month period. In the subgroup analysis looking at specific treatment type, mean haemoglobin was 3.62 g/L lower in those receiving PI-based cART compared with those receiving ZDV monotherapy (*p=*0.002); no such effect was observed for NVP-based cART compared with ZDV monotherapy (*p=*0.82). There was no difference in absolute neutrophil count between NVP-based cART, PI-based cART and ZDV monotherapy-treated infants at one, two or six months of age in the unadjusted analysis; this finding was maintained in the multivariable model adjusting for visit number, ethnicity and gestational age.

**Figure 2:**
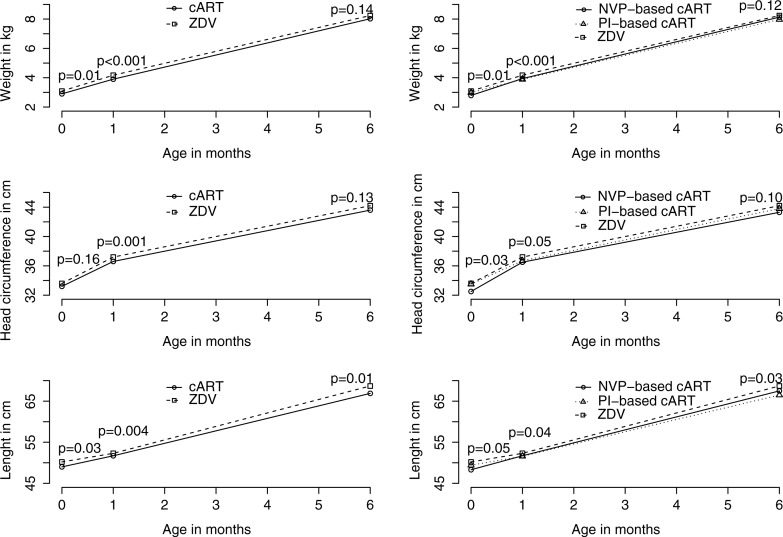
Growth parameters according to treatment group. **Graphic representation of univariate analyses (Student's t-test, Wilcoxon rank-sum, or Kruskal-Wallace test where appropriate.)**

Weight, length and HC were compared between all cART-treated and ZDV-treated infants, and between PI-based cART, NVP-based cART and ZDV at birth, one and six months of age (Table 2 and Figure 2). In the unadjusted analysis, cART-treated infants were smaller compared with ZDV-treated infants at birth (weight 2.91±0.60 vs. 3.09±0.56 kg, *p=*0.01; length 49.0±3.6 vs. 50.2±3.8 cm, *p=*0.03, head circumference 33.2±2.4 vs.33.6±2.3 cm, *p=*0.16). By six months of age, there was no difference in weight (8.03±1.12 kg vs. 8.25±1.12 kg, *p=*0.14) or head circumference (43.7±1.8 cm vs. 44.0±1.7, *p=*0.13), although the cART-treated group remained shorter than ZDV-treated infants (67.1±2.9 cm vs. 68.1±3.2 cm, *p=*0.01). In the subgroup analysis looking at specific treatment type, both NVP-and PI-treated infants were smaller than ZDV-treated infants across all growth parameters at birth; only the difference in length remained significant at six months of age.

To study the overall effect of treatment type on growth parameters over time, we constructed a multivariate fixed effects model adjusting for birthweight, gestational age, chronological age and gender. Overall, there was a mean HC difference of −0.63 cm among cART compared with ZDV-treated infants over the six-month period (*p*<0.001) (Supplementary Table, not shown). A similar effect was seen with respect to weight and length, with a mean weight difference of −236 g and length difference of −0.92 cm over the six-month period (*p<*0.001 and *p=*0.002, respectively). In the subgroup multivariate analysis comparing PI-based cART versus ZDV and NVP-based cART versus ZDV, there was no discernable difference between the two cART treatment types and these growth parameters.

## Discussion

In this retrospective study, cART initiated empirically for newborns deemed at high risk for perinatal HIV infection was generally well-tolerated, though adverse effects and consequent premature discontinuation of therapy did occur more often in the cART versus ZDV-treated group (9.5% vs. 2.1%). Among the 14 cART-treated children in whom medications were prematurely discontinued, four had their medications stopped by the parents without consultation with the healthcare provider and in the absence of any side effects, and six for precautionary reasons by the clinician even though the symptoms were not thought to be medication related, highlighting a lower threshold for treatment discontinuation by both parents and physicians among cART-treated infants.

With respect to haematological toxicity, the frequency and degree of anaemia observed in our cART-treated neonates was higher than that observed in the ZDV-monotherapy neonates. In the subgroup analysis of cART-treated infants, haemoglobin was lower at one month of age among PI versus NVP-treated infants, suggesting perhaps less haematological toxicity with NVP-based cART. The lower mean haemoglobin observed in cART-treated subjects compared with ZDV-monotherapy-treated subjects at one month of age was no longer evident at two and six months of age after stopping treatment, confirming the reversible nature of the anaemia. Reassuringly, compared with ZDV monotherapy, the use of cART was not associated with any discernible change in neutrophil count.

While in the multivariate mixed effects model cART-treated infants had lower mean growth measures (HC, weight and length) than ZDV-monotherapy-treated infants over time, there were no absolute differences in growth measures for head circumference or weight at six months of age, indicating good catch-up growth among the cART-treated infants. The observed lower mean growth over time in cART recipients was therefore likely due to factors associated with the indications for the use of cART. Specifically, the higher overall incidence of prematurity among cART-treated infants was likely a result of poorly controlled maternal HIV, an established risk factor for low birthweight and preterm delivery [17]. The higher viral load and lower CD4 counts of mothers of cART recipients in our study supports this hypothesis. While we adjusted for preterm delivery and gestational age in the multivariate models for growth, we were unable to adjust for *in utero* drug exposure, social situation, maternal health, nutritional status and education, which may be important predictors of infant growth and development.

Because of the selective use of cART for infants at higher risk of perinatal transmission in our study, it is not appropriate to compare the risk of transmission among cART versus ZDV-treated infants as the latter group would have received better antenatal preventive management. The 8.8% overall transmission rate observed among cART recipients in our cohort was similar to that in a previous trial in which neonates of untreated mothers were randomized to different cART regimens, evidence of the high risk of transmission despite prophylaxis in these situations [6]. Key potential benefits of cART at treatment doses initiated as prophylaxis for neonates later found to be infected is that such treatment will preserve health by controlling viral replication more rapidly, reduce the risk of generating antiretroviral medication resistance, as seen with single or multiple dose NVP in non-cART strategies, and limit the size of HIV viral reservoirs, which could have significant implications for achieving HIV remission in the future [18–20]. Given the limited number of therapeutic drug regimens available for children in resource-limited settings, preventing drug resistance to first-line therapy given to prevent perinatal transmission is a particularly important consideration.

Our study has a number of limitations, including its retrospective nature, relatively small sample size, and potential differences in the patient population at different study sites which may have influenced biological and growth outcomes. All control group infants were selected from a single centre, and the choice of PI-based versus NVP-based cART was also site specific. Given that these were the comparison groups of interest, we could not adjust for site and treatment type in the final analysis, thereby limiting our conclusions on specific cART treatment type.

## Conclusions

In summary, our study shows that cART at treatment doses administered as prophylaxis to “high-risk” HIV-exposed neonates is generally well tolerated and should be considered for newborn infants deemed at high risk of perinatally acquired HIV infection. However, close monitoring for anaemia, neutropenia and other side effects is important if such therapy is given. Until more robust pharmacokinetic data are available, the use of cART as prophylaxis in neonates may be limited to resource-rich settings where routine therapeutic drug monitoring is possible. However, further study is urgently needed in resource-limited settings where the vast majority of high-risk HIV-exposed infants are born. The potential benefits of cART, both to avert infection and, in case of transmission, preserve future therapeutic options and potentially limit the viral reservoir, may well outweigh the risks. Finally, if this strategy is used, counselling of parents and close follow-up is necessary to avoid premature treatment discontinuation and to ensure proper monitoring of cART-exposed infants.

## Supplementary Material

Safety of combination antiretroviral prophylaxis in high-risk HIV-exposed newborns: a retrospective review of the Canadian experience
