# Supplementary material for: Safety of combination antiretroviral prophylaxis in high-risk HIV-exposed newborns: a retrospective review of the Canadian experience
Source: J Int AIDS Soc. 2016 Feb 12;19(1):20520. doi: 10.7448/IAS.19.1.20520 (PMC4753845; doi:10.7448/IAS.19.1.20520)
Supplement: Safety of combination antiretroviral prophylaxis in high-risk HIV-exposed newborns: a retrospective review of the Canadian experience [file JIAS-19-20520-s001.pdf]

Supplementary Table: Multivariate model effect size estimates

Parameter	Effect	Estimate	95% CI	P-value
Hemoglobin (g/L) *				
	cART vs ZDV	-2.07	-4.08; -0.06	0.04
	NVP-based cART vs ZDV	0.30	-2.38 ; 2.99	0.82
	PI-based cART vs ZDV	-3.62	-5.89 ; -1.34	0.002
Neutrophil count ($x10^6$/L) **				
	cART vs ZDV	-0.005	-0.206; 0.195	0.95
	NVP-based cART vs ZDV	0.014	-0.248 ; 0.276	0.91
	PI-based cART vs ZDV	-0.020	-0.253 ; 0.220	0.87
Head Circumference (cm) ++				
	cART vs ZDV	-0.63 cm	-0.95 ; -0.30	0.0002
	NVP-based cART vs ZDV	-0.8 cm	-0.9 ; -0.1	0.007
	PI-based cART vs ZDV	-0.5 cm	-1.2 ; -0.4	0.0003
Weight (grams)++				
	cART vs ZDV	-0.283	-0.426 ; -0.140	0.0001
	NVP-based cART vs ZDV	-0.254	-0.445 ; -0.062	0.01
	PI-based cART vs ZDV	-0.302	-0.466 ; -0.138	0.0003
Length (cm) ++	cART vs ZDV			
		-0.92	-1.51 ; -0.33	0.002
	NVP-based cART vs ZDV	-0.7	-1.5 ; 0.1	0.07
	PI-based cART vs ZDV	-1.0	-1.7 ; -0.4	0.003

*** Adjusted for gestational age and visit**

**** Adjusted for gestational age, ethnicity, visit**

++ Adjusted for birthweight, gestational age, visit and gender
